# Heightened Stress in Employed Individuals Is Linked to Altered Variability and Inertia in Emotions

**DOI:** 10.3389/fpsyg.2020.01152

**Published:** 2020-06-16

**Authors:** Diana Wang, Stefan Schneider, Joseph E. Schwartz, Arthur A. Stone

**Affiliations:** ^1^Center for Self-Report Science, University of Southern California, Los Angeles, CA, United States; ^2^Stony Brook Medicine, Stony Brook, NY, United States

**Keywords:** emotion dynamics, perceived stress, aging, variability, affect, inertia

## Abstract

Stress has been widely recognized as a key factor contributing to health outcomes and psychological well-being. While some growing evidence points to stress as having an effect on emotion dynamics characteristics, there has yet to be a test of how global perceptions of stress are associated with not only average levels of emotions but also the variability in the intensity of the emotions, as well as how emotions linger (inertia), and whether these characteristics differ by age. In an effort to better understand how stress influences the emotional experiences of individuals, we examined associations between perceived stress levels and emotion dynamics indices in a sample of 859 working individuals over 24 h. Participants ranged in age from 21 to 81 years. Each participant was prompted at approximately 28 min intervals throughout a 24 h period to report intensity of emotional states. Overall, individuals who were more stressed experienced lower mean levels of positive emotions (with the exception of higher levels of excitement) and higher mean levels of negative emotions. They also experienced more pronounced variability in both positive and negative emotions, and greater inertia in negative emotions. We also found some evidence for age-related differences in mean levels and variability in certain emotions. The relationship of emotion dynamics indices to stress levels was not moderated by age. Many of the stress–emotion dynamics associations did not remain statistically significant upon controlling for the mean level of momentary emotions, indicating that the mean is a large component in the association.

## Introduction

The overall levels of emotions that an individual experiences are important indicators of psychological health status. However, emotions can change over time at the scale of seconds to hours, and a large body of literature has demonstrated that characteristics of emotion fluctuations (emotion dynamics) are important indicators of psychological health (Houben et al., 2015). Therefore, it is important to understand the various psychological and behavioral factors that contribute to emotion dynamics. One’s overall perception of stress has been identified as a key psychological factor that alters emotion dynamics (Koval and Kuppens, 2012). However, evidence of the associations between stress and emotion dynamics to date has been limited to laboratory-induced stressors, which may suffer from a lack of ecological validity in terms of their generalizability to individuals’ real-world perception of stressors (Dickerson and Kemeny, 2004). Higher levels of perceived stress have been shown to be a strong predictor of worse mental health (Keller et al., 2012), accelerated cellular aging (Epel et al., 2004), increased risk of disease (Cohen et al., 2007), and premature mortality (Keller et al., 2012).

As part of an effort to understand the potential pathways through which perceived stress can influence health and well-being, it is of interest to know whether an elevated level of perceived stress is related to the emotional experiences in the daily lives of individuals. Advances in ecological momentary assessment (EMA) methodology allow for the collection of high-resolution data in naturalistic settings as well as reduction in recall bias (Stone and Shiffman, 1994). To date, the role of global perceptions of stress has yet to be examined in association with short-term emotion dynamics during everyday life. Further, aging-related theory and empirical evidence of reactivity to daily stressful events suggest that perceptions of stress might be *differentially* associated with emotion dynamics over the adult life course (Charles, 2010; Scott et al., 2013). In the present study, we examined associations of perceived stress levels and age with emotion dynamics, and whether age moderates the associations between levels of perceived stress and emotion dynamics. Finally, a recent meta-analysis reported that psychological well-being states did not predict emotion dynamics indices above and beyond the mean levels of momentary emotional states (Dejonckheere et al., 2019a). Thus, we set out to examine whether our hypothesized associations hold after controlling for the mean levels of emotions, as this would aid in our understanding of the extent to which the emotion dynamics indices are independent of mean levels of an individual’s emotions.

While there is a wide array of emotion dynamics indices, we focus on two primary measures: variability (innovation variance) and inertia (autoregressive parameter). The level of an emotional state depends in part on the previous level of an emotion as well as a number of other factors that occur, such as negative events, or social interactions (Jongerling et al., 2015). *Innovation variance* reflects the proportion of emotion fluctuations that are not predicted from the previous emotional states, and are therefore considered to be due to exposure and/or reactivity to events. *Emotional inertia* refers to the ability of the intensity of an emotional state at one moment to predict the intensity measured at a subsequent moment. This is measured by a first-order autoregressive parameter (AR) of emotions across time, with a higher inertia value indicating that an emotion lingers longer (Houben et al., 2015). In these analyses, we use dynamic structural equation modeling (DSEM), which allows for between-person variability in innovation variances through including a random effect for the parameters. It is important to note that many previous studies have used summary statistics such as standard deviation to examine net variability, which is a function of both the innovation variance and autoregressive parameter, whereas the DSEM modeling approach offers a novel method to examine these constructs. Temporal dynamics indices of emotional variability and inertia have been linked to psychological health: a recent meta-analysis found greater variability and greater inertia in emotions in individuals with low psychological well-being and in those with psychiatric disorders (Houben et al., 2015).

Hypotheses advanced in this study are largely based on findings from studies that have found that variability and inertia of emotions are altered at various stages of a stressful experience. For example, depressed adolescents experienced higher inertia in emotional behavior during experimentally induced stressful interactions with their parents (Kuppens et al., 2010). In another study, individuals who were exposed to a laboratory stressor experienced a decrease in inertia of emotional states in anticipation of the stressor. Paradoxically, those who are particularly sensitive to negative evaluation by others generally experience higher levels of inertia overall, but experience a larger drop in inertia in anticipation of a stressor (Koval and Kuppens, 2012). These studies demonstrate that short-term exposure to laboratory stressors alters emotional inertia, though the direction in which inertia changes seems to vary based on individual traits, as well as timing. There is also evidence that stress can impact variability in emotions. In younger adults who experienced a breakup, variability in both positive and negative emotions was heightened in the week following the breakup (Sbarra and Emery, 2005). While these studies show that emotion fluctuations may be altered before, during, and after the experience of a stressor, it is also important to understand how stress as it is generally perceived in one’s life – that is, people who are generally feeling high levels of stress versus those who do not – is associated with emotion dynamics. We hypothesize that those who perceive a higher level of global stress compared to those with lower levels will have higher levels of variability and inertia in emotional states.

Furthermore, other dimensions of emotional experience have been shown to change under stress. Empirical evidence demonstrates a reduction in the amount with which positive and negative emotions are differentiated on stressful days and following a stressful event (Zautra et al., 2002, 2005). More recently, it was found that during what may be considered an acute stressful life event of receiving college examination results, individuals’ positive and negative affect shifted from having a weaker negative correlation to having greater bipolarity (Dejonckheere et al., 2019b). Finally, the fluctuations in the differentiation between emotions differ such that differentiation of negative emotions (the ability to identify emotions with specificity) was lower when individuals were experiencing higher levels of stress (Erbas et al., 2018). Taken together, these findings demonstrate that the experience of stressful events is associated with shifts in the differentiation between negative and positive affect; stress not only influences processes that underlie temporal dynamics, but also the space in which emotions vary.

As demonstrated by recent empirical findings, emotion dynamics also change with age, and several theoretical perspectives suggest developmental changes in emotion regulation that may underlie these trends. In one study in which individuals’ emotions were assessed daily over 45 days, older adults (ages 70–80) had less variability (lower intraindividual standard deviations) in both positive and negative emotional states compared to younger adults (ages 20–30) after controlling for mean affect level differences (Röcke et al., 2009). In another study, older adults (ages 65–80) were found to have lower variability in emotional states that were measured once a day over 100 days, compared to younger adults (ages 20–31) (Brose et al., 2013). In a 7-day ecological momentary assessment (EMA) study in which emotional states were measured five times a day, this negative association between age and emotion variability was found for both positive and negative emotions within a day (Carstensen et al., 2000, 2011). Based on these findings, we predicted that older individuals would have lower levels of variability in emotions. There is much less evidence for the association between age and inertia of emotions, though when compared with younger adults (aged 20–31), older adults (aged 65–80) have higher inertia in positive emotions, and lower inertia in negative emotions (Hamaker et al., 2018). Given the limited evidence for age differences in inertia, our analyses of age–inertia associations were exploratory in nature.

Theoretical perspectives also suggest that age may moderate the association between stress and emotion dynamics indices. The Strength and Vulnerability Integration (SAVI) model posits that there are age-related improvements in strategies to avoid or reduce exposure to distress, such that older adults respond better emotionally than younger adults to distressing situations (Charles and Carstensen, 2008; Charles, 2010). However, the age-related advantage in regulating emotions is reduced under circumstances in which people cannot easily use these skills, such as the continued exposure to chronic unrelenting stressors. The advantage that older adults seem to have in emotion regulation is hypothesized to exist only in the context of lower levels of perceived stress. Based on the SAVI model, we also examined the interaction between age and stress in predicting variability and inertia of emotions. We hypothesized that while older age will be associated with lower variability of emotions, this relationship will be less pronounced for individuals who report higher levels of perceived stress. As inertia (in particular, of negative emotions) has been thought to reflect poor emotion regulation capacity, we hypothesize that there will also be an interaction between age and perceived stress levels, such that older adults would have greater inertia of negative emotions in the context of higher levels of stress, and less inertia when stress levels are low.

The majority of studies on emotion dynamics were based on end-of-day reports or measurements taken a few times throughout a day, which yield data with recall periods over 24 h, or at best, over several hours. Since shifts in emotional states can occur quickly (on the scale of seconds to minutes), shorter intervals between measurements may provide an entirely different picture of the emotional lives of individuals. In EMA research, there is often a balance in the frequency of measurements throughout the day and the number of days sampled, in efforts to manage the participant burden. Given the existing evidence that short-term emotion fluctuation processes are appropriately measured using 15 or 30 min measurement increments (Ebner-Priemer and Sawitzki, 2007), our research question was better suited to examine emotions measured in higher frequency under a shorter measurement period, as opposed to emotions measured every 2–4 h, or at the end of the day. While acknowledging that a 24 h period may be less ideal than having a sample of multiple days, the processes that we are aiming to capture with the emotion dynamics indices are best examined in this frequency. The current study utilized data from the Masked Hypertension Study (MHTS), which assessed momentary emotions at a high density (every 28 min), allowing for the analysis of short-term fluctuations in emotional states. With this dataset, we set out to examine whether perceived stress and age were associated with more or less variability and inertia in emotions, and whether age and stress interact to predict these emotion dynamics.

Our study also utilizes an innovative statistical approach to model indices of emotion dynamics – dynamic structural equation modeling (DSEM) (Hamaker et al., 2018). This method allows us to model individual differences in innovation variance, autoregressive parameter (inertia), and mean affect levels, as latent (i.e., random) variables in multilevel models. Thereby, the DSEM method sampling error in these indices (which results from the obtained momentary reports being only a random sample of all possible reports that could have been obtained from each participant) further provides an elegant solution for unequal spacing between measures that often occurs in EMA studies due to missing data (Hamaker et al., 2018).

## Materials and Methods

### Sample

Data from the MHTS, a multi-site study conducted at Stony Brook University and Columbia University in 2005–2012, were used in this study. The primary goal of the MHTS was to examine the phenomenon of masked hypertension and related psychosocial factors. To be eligible for the study, participants had to be age 21 or older and employed at either of the universities or a financial institution in the New York City metropolitan area. Recruitment criteria included a screening blood pressure of below 160/105 mmHg, and those who were using medication that lowered blood pressure were excluded from the study. Participants were also excluded if they had evidence of secondary hypertension, a history of overt cardiovascular disease, chronic renal, liver, thyroid, or adrenal disease, or cancer not in remission for at least 6 months, active substance abuse, or a serious mental health illness. A total of 1,011 participants were consented and enrolled in the MHTS. The study was approved by the Institutional Review Boards (IRBs) of Stony Brook University and Columbia University. The University of Southern California IRB approved the secondary analyses reported here.

In the MHTS, 903 participants completed EMAs of emotional states throughout a 24 h period. Participants were provided with a pre-programmed electronic diary (Palm Pilot Tungsten 3), on which they were prompted to answer EMA questions about their situation, activities, emotional states, and social interactions immediately prior to ambulatory blood pressure measurements that were taken approximately 28 min apart over a 24 h period that included one full or parts of two workdays (Schwartz et al., 2016).

### Measures

#### Emotional States

Ecological momentary assessments of emotional states included items that measured positive and negative emotions on a horizontal visual analog scale using anchors at 0 (Not at all) and 100 (Very much). Participants were presented with a question stem of “Just before [the] BP [reading]: How ____ were you feeling?” where BP referred to the ambulatory blood pressure reading that served as the signal to complete an electronic diary entry. Positive emotional state items included “excited,” “happy,” and “relaxed.” Negative emotional state items included “frustrated,” “angry/hostile,” “anxious/tense,” and “depressed/blue.”

#### Global Perceived Stress

Participants completed the Perceived Stress Scale (PSS), which addresses the extent to which in the last month, an individual perceives current life demands as uncontrollable or overwhelming and how well they believe they can deal with it (PSS; Cohen et al., 1983). Participants completed the 14-item PSS as part of the psychosocial questionnaire within 2 weeks before the EMA assessments. Example items include: “In the last month, how often have you felt difficulties were piling up so high that you could not overcome them?” Participants were asked to provide their responses on a 5-category Likert scale of “Never” to “Very often.”

### Analytical Plan

We used DSEM in M*plus* version 8.3 (Muthén and Muthén, 2017) to examine the associations between both inter-individual factors (age and stress levels) and the intra-individual mean level, variability, and inertia of the emotional states (Mcneish and Hamaker, 2017). We examined each of the emotion items assessed in the study in separate models (as opposed to summary scores of positive emotions and negative emotions, for example).

**FIGURE 1:**
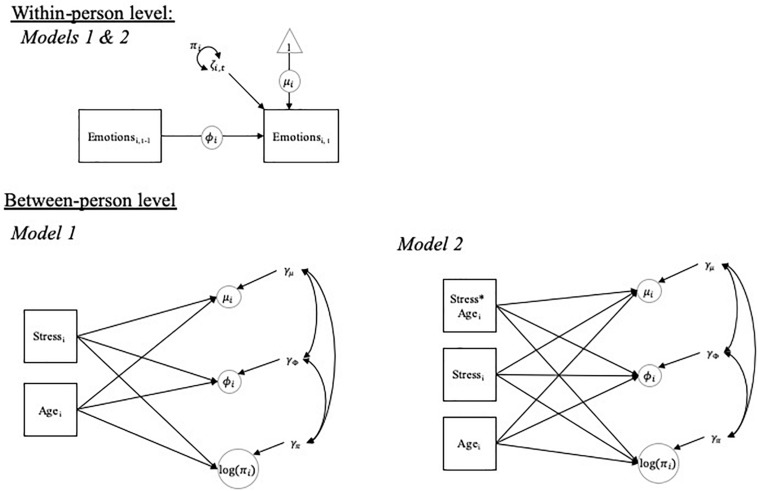
Multilevel DSEM model.

Multilevel DSEM is based on decomposing the data into a within-person and between-person part (illustrated in Figure 1). On the within-person level, the momentary emotional state for person *i* at time point *t* (*Emotion*_i,t_) is regressed on the preceding state for the same emotion at time point *t*-1 (*Emotion*_i,t__–__1_) in a time-series model. The resulting autoregressive parameter φ_i_ assumes values between −1 and 1, where more strongly positive values indicate that it takes a person longer to return back to his or her “normal” state (i.e., the person shows more inertia) after being perturbed; this “normal” state is represented by the person’s mean emotion level μ_i_. The residual deviations of emotional states from the person’s mean (ζ_i,t_) have a variance of π_i_, where this variance represents the magnitude of emotion variability within the person. All parameters have a subject index *i* to indicate that the within-person mean level (μ_i_), variability (π_i_), and inertia (φ_i_) of an emotion item can differ from person to person.

On the between-person level, individual differences in each of these three parameters are represented as latent variables (shown as circles on the between-person level in Figure 1) that are modeled as multivariate outcomes (i.e., they are allowed to correlate with each other). Random effects for the within-person mean (μ_i_) and inertia (φ_i_) parameters are assumed normally distributed, whereas the random effect for within-person variability (π_i_) is assumed to follow a log-normal distribution (i.e., the within-person variance is implicitly log-transformed to normalize its distribution). In the models, the random effects in means, variability, and inertia were regressed simultaneously on both perceived stress and age (Model 1 in Figure 1). Then, the interactions between perceived stress and age were examined in predicting each of the parameters (Model 2 in Figure 1).

The DSEM implementation in M*plus* is based on Bayesian parameter estimation using a Markov chain Monte Carlo (MCMC) algorithm. We used the potential scale reduction criterion (Gelman et al., 2013) to decide on the number of MCMC iterations needed for convergence of each model. We present regression estimates along with 95% credible intervals provided in Bayesian analysis (these can be interpreted analogous to 95% confidence intervals).

### Time Intervals

The DSEM method addresses the challenge presented by unequal spacing between observations. In the present study, respondents were signaled every 28 min to complete the EMA ratings. However, individuals did not provide measures of emotional states overnight when they were asleep, in addition to missing prompts for other reasons throughout the day, resulting in unequal time intervals (gaps) between observations. These are especially problematic for the estimation of autoregressive parameters (i.e., inertia) because the size of the parameter depends on the length of the lag. In M*plus*, this problem is approached by dividing the person’s day into 28 min segments and inserting a missing value into any time segment for which there is no observation (i.e., no EMA rating). The missing values are treated as missing at random, and the method has been shown to yield appropriate parameter estimates in Bayesian analysis even when a large amount (80%) of missing values is inserted (Asparouhov et al., 2018).

## Results

The sample was restricted by excluding those who did not have data on perceived stress levels or age. This resulted in a total of 859 participants. The mean age for the sample was 44.8 years (*SD* = 10.4, range 21–81 years). The sample is 59% female, 7.4% Black/African American, and 12% Hispanic. The mean perceived stress level was 21.74 (ranging from 0 to 51), which is comparable to previously reported values of 23 in two United States samples (Cohen et al., 1983; Cohen and Janicki-Deverts, 2012). The average compliance rate was 76.3% (*SD* = 18.0, median = 80.5%) for this sample. Momentary emotional state scores were divided by 10 causing the transformed scores to range from 0 to 10. For the positive valence emotional states, participants reported an average of 2.1 in levels of excitement, 5.3 in happiness, and 5.3 in relaxation (see Table 1). They reported substantially lower mean levels of negative emotions, with a mean level of 1.5 in anxiety, 1.4 in frustration, 0.5 in depressed, and 0.8 in anger. The average variability (log variance) of the emotional states ranged from −2.3 (relaxation) to 1.1 (depressed), and average inertia (autocorrelations over 28 min) ranged from 0.27 (excitement) to 0.41 (depressed).

**TABLE 1:** Descriptive statistics (mean and SD) of individual differences in mean levels, variability, and inertia of each emotional state.

Emotional state	Mean emotion level^a^	Variability^b^	Inertia^c^
Frustration	1.360 (0.916)	0.113 (1.856)	0.308 (0.240)
Anxiety	1.528 (1.114)	−0.072 (1.849)	0.348 (0.246)
Depression	0.505 (0.437)	−2.262 (2.530)	0.321 (0.307)
Anger	0.758 (0.601)	−1.130 (2.356)	0.270 (0.246)
Excitement	2.086 (1.446)	0.351 (1.601)	0.291 (0.211)
Happiness	5.253 (1.646)	0.364 (0.967)	0.386 (0.222)
Relaxation	5.322 (1.421)	1.091 (0.733)	0.413 (0.217)

*^a^Person mean level on a 0–10 scale. ^b^Log intraindividual variance. ^c^First order autocorrelation; Estimates come from an empty DSEM model which does not include any predictors.*

Results from analyses examining perceived stress and age as predictors of the mean level, variability, and inertia of each emotional state are presented in Table 2. We first summarize the perceived stress associations. We found that those who reported higher levels of global perceived stress experienced significantly higher mean levels of negative emotions and lower mean levels of positive emotions; an exception, however, was that higher global stress was associated with higher levels of the positive emotional state of excitement. Higher levels of perceived stress were also significantly associated with greater variability in both negative and positive emotional states (with the exception of a non-significant association with the variability in relaxation). Higher levels of stress were also significantly associated with higher inertia in negative emotional states. No significant association was found between stress and inertia in the three positive emotional states (excitement, happiness, and relaxation).

**TABLE 2:** Effect sizes of perceived stress and age on mean, variability, and inertia of each emotional state.

	Mean emotion level^a^			Variability^b^			Inertia^c^		
	*B*	95% CI	*R*^2^	*B*	95% CI	*R*^2^	*B*	95% CI	*R*^2^
**Frustration**									
Stress	0.222	0.175, 0.264	0.075	0.329	0.249, 0.410	0.044	0.039	0.026, 0.053	0.034
Age	–0.069	−0.129, −0.001		–0.191	−0.304, −0.070		0.006	−0.013, 0.026	
**Anxiety**									
Stress	0.274	0.218, 0.325	0.072	0.365	0.283, 0.446	0.047	0.039	0.025, 0.053	0.031
Age	–0.008	−0.079, 0.070		0.017	−0.094, 0.137		0.005	−0.016, 0.023	
**Depression**									
Stress	0.100	0.079, 0.121	0.062	0.534	0.426, 0.642	0.054	0.066	0.051, 0.080	0.060
Age	0.020	−0.007, 0.051		0.029	−0.122, 0.190		0.001	−0.020, 0.023	
**Anger**									
Stress	0.232	0.180, 0.279	0.058	0.200	0.155, 0.247	0.043	0.152	0.093, 0.210	0.027
Age	–0.029	−0.077, 0.025		–0.042	−0.087, 0.006		–0.036	−0.097, 0.023	
**Excitement**									
Stress	0.091	0.023, 0.156	0.029	0.133	0.063, 0.204	0.019	0.002	−0.012, 0.017	0.005
Age	–0.296	−0.386, −0.197		–0.212	−0.321, −0.113		–0.017	−0.036, 0.001	
**Happiness**									
Stress	–0.361	−0.438, −0.285	0.058	0.066	0.023, 0.111	0.016	0.001	−0.013, 0.015	0.002
Age	0.043	−0.063, 0.148		–0.128	−0.192, −0.067		–0.003	−0.022, 0.017	
**Relaxation**									
Stress	–0.316	−0.380, −0.254	0.065	0.032	−0.001, 0.068	0.003	0.007	−0.006, 0.021	0.003
Age	0.125	0.024, 0.229		–0.012	−0.063, 0.041		–0.007	−0.025, 0.012	

*^a^Person mean level of emotion is on a 0–10 scale. ^b^Log intraindividual variance. ^c^First order autocorrelation. In these models, to allow for convenient interpretability and convergence of DSEMs, we transformed independent variables by dividing age by 10, and perceived stress scores by 5.*

Turning to age and emotions, we found that older age was significantly, yet weakly, associated with lower mean levels of excitement and higher mean levels of relaxation. Older age was also significantly but weakly associated with less variability in frustration, excitement, and happiness. There were no associations between age and inertia of emotions. Finally, we observed no significant interactions of age with perceived stress when predicting mean levels, variability, or inertia of emotions (results not shown; available on request). Together, age and stress accounted for between 5.8 and 7.5% of the variation in the mean levels of frustration, anxiety, depression, anger, happiness, and relaxation, and 2.8% of the variation in mean excitement. Of the emotion dynamics indices, they accounted for 4–5% of the variation in the variability of the negative emotional states, but under 2% in that of negative emotional states. Age and perceived stress, primarily the latter, accounted for approximately 3% of the variation in inertia of frustration, anxiety, and anger, 6% of the variation in inertia of depression, and less than 1% of that in positive emotional states.

A question that is inevitably raised in investigations of emotion dynamics is whether the associations of indices of variability and inertia with the variables of interest (in our case, age, and perceived stress) might be spurious, attributable to their shared associations with the mean emotion level. Indeed, in our sample, mean levels of emotions exhibit sizeable correlations with the variability and inertia indices, especially for the negative emotions (see Table 3). We ran supplementary DSEM analyses in which the mean levels of emotion were included as a potential mediator in the equations predicting the variability and inertia of emotions. We found that the independent effect of perceived stress on the variability of affective states, after controlling for mean levels, became non-significant for frustration, depression, anger, and happiness, but not for anxiety and excitement (see Supplementary Table 1). Similarly, the effect of independent perceived stress on the inertia of negative affective states became non-significant for frustration and anxiety, but not for depression and anger, after controlling for mean levels. The indirect effects of stress on variability and inertia via mean levels of affective states were significant in all instances, suggesting that the mean level of momentary emotions is a large underlying component in the association between perceived stress and emotion dynamics. An additional way to examine the associations of intra-individual factors (age, perceived stress levels) with mean levels, variability, and inertia while controlling for the correlations among the emotion dynamics indices is simultaneously regressing the emotion dynamics indices onto age and perceived stress. These results mirror those from the models controlling for the mean, demonstrating remaining associations between higher levels of stress and greater variability in anxiety and excitement, and greater inertia in depressive symptoms (see Supplementary Table 2).

**TABLE 3:** Correlations among mean levels, variability, and inertia for each emotional state.

Emotional state	Correlation mean – variability	Correlation mean – inertia	Correlation variability – inertia
Frustration	0.688	0.623	0.372
Anxiety	0.664	0.533	0.352
Depression	0.885	0.783	0.661
Anger	0.759	0.704	0.454
Excitement	0.563	0.439	0.325
Happiness	–0.294	–0.001	–0.108
Relaxation	–0.206	–0.008	–0.314

## Discussion

Emotions fluctuate and the fluctuations are thought of as outputs from an affective system that responds to both external events and internal regulatory processes (Kuppens et al., 2010). In this study we examined whether perceived stress and age were independently predictive of mean, variability, and inertia of several emotional states. As expected, individuals with higher levels of global perceived stress experienced higher mean levels of negative emotions and excitement, and lower mean level of happiness and relaxation. In addition, we found that those with higher perceived stress exhibited greater variability in emotions throughout the day, and that negative emotions tended to linger for longer in those with higher perceived stress levels. There was no consistent evidence that the variability or inertia differed across age, but our data do suggest that variability in frustration, excitement, and happiness was lower in older individuals. Finally, we hypothesized that there would be an interaction between age and stress in predicting emotion dynamics, but did not find evidence for this.

### Associations Between Stress and Mean, Variability, and Inertia of Emotional States

While it may be a reasonable expectation that individuals with more perceived stress experience all positive affective states at lower levels, we did find higher mean levels of excitement in those who are more stressed. A potential explanation for this may be that both stress and excitement are high activation or high arousal emotions, and as some theoretical perspectives would suggest, they are likely to occur together (Watson and Tellegen, 1985; Posner et al., 2005). Consistent with our hypotheses, we also found greater variability in emotions across both positive and negative valence emotions in those who are more stressed. Greater variability in emotions has been consistently found in those with psychopathology and poorer psychological well-being in nonclinical populations (Houben et al., 2015). Importantly, this is in contrast to the interpretation of findings from much of the existing literature which has used more traditional calculation methods of SD as variability. By using the DSEM method, we have been able to model the innovation variance, thereby explicitly allowing for individual differences in unobserved shocks to the emotion regulation system beyond what can be predicted by the level of intensity of a preceding emotional state.

We now turn to the last emotion dynamics index that we examined, inertia. Some have suggested that greater inertia of emotions reflects ineffective emotion regulation capacity in response to events, although it is inconclusive whether inertia predominantly reflects *internal* emotion regulation processes or more prolonged exposure to *external* circumstances (Kuppens and Verduyn, 2015). On the one hand, greater inertia in negative emotions has been linked to the tendency to suppress the expression of feelings (Koval et al., 2015b) as well as rumination (Koval and Kuppens, 2012). Some evidence suggests that inertia reflects exposure to more intense events, but not the frequency of events (Koval et al., 2015a). On the other hand, higher inertia has also been associated with impaired *recovery* from emotional stimuli in the lab, though this association was weak and requires replication (Koval et al., 2015a). Longitudinal research is needed to clarify whether chronically elevated levels of perceived stress might be related to altered emotion fluctuations during daily life.

### Associations Between Age and Mean, Variability, and Inertia of Emotional States

We found some evidence that age was associated with mean levels and variability of emotional states. To aid in the interpretation of the results, we refer to the Circumplex Model of Affect (Posner et al., 2005). According to this model, the emotional states measured in this study can be categorized into positive valence high arousal (excitement, happiness), positive valence low arousal (relaxed), negative valence high arousal (anger, frustration, anxiety), or negative valence low arousal (depression). While the strengths of the associations are weak, our findings suggest that older adults experience relatively lower *mean* levels of the high-arousal state of excitement, and higher mean levels of the low-arousal relaxation. Older age also predicted lower *variability*, particularly in the high-arousal emotions of frustration, excitement, and happiness. Our findings demonstrate the importance of examining individual emotional state items, and pursuing further research on age-related shifts in the arousal dimension of emotional states. Aging and developmental theories suggest a decrease in negative valence emotions, and increase in positive valence emotions as people age (Carstensen and Mikels, 2005). The average age of participants was relatively young at 45 years (range 21–81 years), and thus, it will be important to extend these analyses to investigate these associations across a sample of individuals that is more evenly distributed across the age range.

Changes in emotion dynamics with older age may be attributed to several factors, including changes in social and environmental contexts. For example, changes in status in the workplace, more familiar routines, or changes in the composition of social networks may lead to greater stability in emotions (Röcke et al., 2018). It is possible that older adults are simply *exposed* to fewer negative events or differing intensity of events compared with middle-aged and younger adults (Almeida and Horn, 2004). Indeed, previous studies have found that those who were older reported fewer stressors, which were less heterogeneous in nature and less disruptive, suggesting that age-related trends in emotion dynamics are related to context (Brose et al., 2013). Although the current study did not examine external factors that may influence the interindividual differences in emotion dynamics indices, it will be important for future research to include environmental factors to determine the extent to which these indices represent endogenous factors. For example, we suggest future studies to collect comprehensive data on the momentary level (event exposure frequency and intensity) as well as global assessments of contextual factors that may account for interindividual differences in emotion dynamics.

The findings from our supplementary analyses are consistent with those of a recent meta-analysis, which shows that many indices do not have independent associations with psychological well-being variables above and beyond mean levels (Dejonckheere et al., 2019a). This calls for caution when examining psychological well-being states and emotion dynamics indices. However, our findings might also suggest that stress affects multiple aspects of emotion dynamics in concert with each other, and more strongly so for some emotions than others.

### Strengths and Limitations

Some strengths of this study are that participants reported their experience of emotions at a high frequency for 24 h, approximately every half hour while participants are awake. The instructions for momentary reporting of emotional states are thought to increase the accuracy of reported information and minimize the biases in retrieval or reconstruction of memory. Much of the existing emotion dynamics research uses end-of-day reports on emotional states, which cannot provide as fine-grained a portrayal of the shifts in emotions. We also chose not to collapse emotions by valence in our analyses, and instead examined each emotional state individually. Our age-related associations illustrate that when considering potential developmental processes, creating sum scores by positive or negative valence may obscure important age differences by high or low arousal. The use of DSEM in modeling the random innovation variance allows for more explicit modeling of inter-individual differences that were previously unexplained with more traditional variability measures. This is particularly important in examining emotional dynamics in relation to perceived stress; we believe that there are individual differences in the unobserved factors (events that occur, behaviors) related to higher perceptions of stress, as well as emotional responsiveness or sensitivity to these factors.

While this study included participants with a wide age range, the average age was relatively young, and all were employed and healthy. The associations that we find are relevant for the working population, but not necessarily generalizable to other populations, such as unemployed or retired individuals. Furthermore, we speculate that the types of stressors that participants were exposed to over the workday and that contributed to perceptions of global stress would more frequently have been related to events at work (compared to the general population). It remains a possibility that age-related changes in emotion fluctuations are due to contextual differences such as changes in social networks, and more research is needed to examine the possibility of contextual differences that occur with aging, or age-related difference in the type/content, frequency, intensity, or appraisal of external events. A systematic examination in a sample which includes more older individuals, including nonworking individuals as well as more data on individuals’ environmental and social contexts would be helpful in teasing apart exogenous (exposure-related) or endogenous (regulation-related) factors that underlie emotion dynamics.

## Summary

These results show that feeling higher levels of stress is associated not only with higher average levels of negative emotions and lower average levels of most positive emotions, but also with the ways in which these emotions fluctuate over the course of a day. Individuals who generally feel more stressed are likely to experience greater variability in both positive and negative emotions, and their negative emotions linger for longer. We also observed a tendency for older individuals to exhibit lower mean levels and variability in high-arousal emotions, which warrants further research into developmental processes using EMA methods. The findings from this study add to our understanding of how perceived stress is associated with altered emotional functioning. While there have been strides toward improving the field’s understanding of the mechanisms through which stress influences physiological functioning as well as emotional reactivity to stressors, this study contributes toward filling a gap in our understanding of how perceived stress may be linked with everyday profiles of emotions, yielding a high-resolution snapshot of the ongoing processes that have an accumulative impact on our health and well-being over time.

## Data Availability Statement

The raw data supporting the conclusions of this article will be made available by the authors, without undue reservation.

## Ethics Statement

The study was approved by the Institutional Review Boards (IRBs) of Stony Brook University and Columbia University. The University of Southern California IRB approved the secondary analyses reported here.

## Author Contributions

DW, AS, and SS contributed to the conception and design of the analyses and wrote sections of the manuscript. JS developed and oversaw the collection of the data. DW and SS performed the statistical analysis. DW wrote the first draft of the manuscript. All authors contributed to the manuscript revision, read and approved the submitted version.

## Conflict of Interest

AS was a Senior Scientist with the Gallup Organization and was a consultant for Adelphi Values. The remaining authors declare that the research was conducted in the absence of any commercial or financial relationships that could be construed as a potential conflict of interest.
