# Supplementary material for: Heightened Stress in Employed Individuals Is Linked to Altered Variability and Inertia in Emotions
Source: Front Psychol. 2020 Jun 16;11:1152. doi: 10.3389/fpsyg.2020.01152 (PMC7309515; doi:10.3389/fpsyg.2020.01152)
Supplement: Supplementary file 1 [file Table_1.docx]

Supplementary Table 1: Associations of Age with emotion dynamics indices when controlling for the mean

|  |  | Variability ^b^ | | Inertia ^c^ | |
| --- | --- | --- | --- | --- | --- |
|  |  | B | 95% CI | B | 95% CI |
| Frustration | Stress | 0.028 | -0.051, 0.103 | 0.002 | -0.012, 0.016 |
|  | Age | -0.095 | -0.195, 0.003 | 0.017 | -0.001, 0.037 |
|  | Mean Frustration | 1.357 | 1.212, 1.508 | 0.164 | 0.135, 0.195 |
|  |  |  |  |  |  |
| Anxiety | Stress | 0.080 | 0.003, 0.154 | 0.007 | -0.008, 0.022 |
|  | Age | 0.028 | 0.290, -0.070 | 0.005 | -0.016, 0.023 |
|  | Mean Anxiety | 0.114 | 0.092, 0.137 | 0.114 | 0.092, 0.137 |
|  |  |  |  |  |  |
| Depression | Stress | 0.038 | -0.033, 0.108 | 0.015 | 0.003, 0.027 |
|  | Age | -0.068 | -0.163, 0.025 | -0.008 | -0.028, 0.009 |
|  | Mean Depression | 4.965 | 4.487, 5.491 | 0.516 | 0.453, 0.586 |
|  |  |  |  |  |  |
| Anger | Stress | 0.065 | -0.017, 0.144 | -0.001 | -0.016, 0.011 |
|  | Age | -0.064 | -0.178, 0.044 | -0.007 | -0.026, 0.012 |
|  | Mean Anger | 2.920 | 2.633, 3.252 | 0.288 | 0.246, 0.337 |
|  |  |  |  |  |  |
| Excitement | Stress | 0.078 | 0.016, 0.140 | -0.004 | -0.019, 0.009 |
|  | Age | -0.026 | -0.121, 0.060 | 0.002 | -0.019, 0.022 |
|  | Mean Excitement | 0.612 | 0.540, 0.681 | 0.067 | 0.051, 0.084 |
|  |  |  |  |  |  |
| Happiness | Stress | 0.008 | -0.037, 0.054 | -0.001 | -0.016, 0.013 |
|  | Age | -0.122 | -0.182, -0.062 | -0.002 | -0.021, 0.017 |
|  | Mean Happiness | -0.163 | -0.212, -0.114 | -0.003 | -0.018, 0.011 |
|  |  |  |  |  |  |
| Relaxation | Stress | 0.000 | -0.039, 0.040 | 0.008 | -0.008, 0.022 |
|  | Age | 0.002 | -0.049, 0.055 | -0.007 | -0.026, 0.013 |
|  | Mean Relaxation | -0.103 | -0.148, -0.053 | 0.001 | -0.017, 0.020 |
