# Supplementary material for: Heightened Stress in Employed Individuals Is Linked to Altered Variability and Inertia in Emotions
Source: Front Psychol. 2020 Jun 16;11:1152. doi: 10.3389/fpsyg.2020.01152 (PMC7309515; doi:10.3389/fpsyg.2020.01152)
Supplement: Supplementary file 2 [file Table_2.docx]

Supplementary Table 2: Associations of each affect dynamic parameter with perceived stress (Regression of mean, variability, and inertia on PSS)

|  |  | Perceived Stress | | Age | |
| --- | --- | --- | --- | --- | --- |
|  |  | B | 95% CI’s | B | 95% CI’s |
| Frustration | Mean | 0.528 | 0.254, 0.780 | -0.134 | -0.316, 0.062 |
|  | Variability | 0.036 | -0.046, 0.119 | -0.046 | -0.105, 0.016 |
|  | Inertia | 0.179 | -0.808, 1.045 | 0.529 | -0.178, 1.104 |
|  |  |  |  |  |  |
| Anxiety | Mean | 0.365 | 0.210, 0.518 | -0.056 | -0.164, 0.062 |
|  | Variability | 0.081 | 0.010, 0.154 | 0.015 | -0.036, 0.070 |
|  | Inertia | 0.338 | -0.344, 0.972 | 0.128 | -0.415, 0.610 |
|  |  |  |  |  |  |
| Depression | Mean | 0.334 | -0.542, 1.076 | 0.530 | -0.039, 1.137 |
|  | Variability | 0.071 | -0.025, 0.171 | -0.058 | -0.130, 0.011 |
|  | Inertia | 0.869 | 0.123, 1.619 | -0.301 | -0.910, 0.240 |
|  |  |  |  |  |  |
| Anger | Mean | 0.646 | 0.081, 1.094 | 0.110 | -0.217, 0.484 |
|  | Variability | 0.056 | -0.016, 0.131 | -0.037 | -0.093, 0.018 |
|  | Inertia | 0.042 | -0.873, 1.110 | -0.341 | -1.132, 0.360 |
|  |  |  |  |  |  |
| Excitement | Mean | 0.056 | -0.047, 0.158 | -0.147 | -0.218, -0.076 |
|  | Variability | 0.106 | 0.024, 0.184 | -0.018 | -0.068, 0.036 |
|  | Inertia | -0.188 | -1.013, 0.649 | -0.027 | -0.647, 0.503 |
|  |  |  |  |  |  |
| Happiness | Mean | -0.301 | -0.363, -0.240 | 0.003 | -0.044, 0.047 |
|  | Variability | 0.020 | -0.099, 0.132 | -0.154 | -0.238, -0.073 |
|  | Inertia | 0.002 | -0.647, 0.625 | -0.101 | -0.554, 0.358 |
|  |  |  |  |  |  |
| Relaxation | Mean | -0.359 | -0.439, -0.285 | 0.077 | 0.019, 0.132 |
|  | Variability | 0.029 | -0.152, 0.198 | -0.004 | -0.123, 0.108 |
|  | Inertia | 0.315 | -0.350, 1.034 | -0.173 | -0.687, 0.298 |
